# Testes Ergométricos em Pacientes com Anemia Falciforme: Segurança, Viabilidade e Possíveis Implicações no Prognóstico

**DOI:** 10.36660/abc.20200437

**Published:** 2022-03-10

**Authors:** Christiano Gonçalves de Araújo, Maria Betânia Solis Resende, Julia Teixeira Tupinambás, Rebeca Coeli Teodoro Maciel Dias, Flávio Coelho Barros, Maria Carmen Melo Vasconcelos, José Nelio Januário, Antonio Luiz Pinho Ribeiro, Maria Carmo P. Nunes

**Affiliations:** 1 Programa de Pós-graduação em Infectologia e Medicina Tropical Faculdade de Medicina Universidade Federal de Minas Gerais Belo Horizonte MG Brasil Programa de Pós-graduação em Infectologia e Medicina Tropical, Faculdade de Medicina, Universidade Federal de Minas Gerais, Belo Horizonte, MG – Brasil; 2 Hospital das Clínicas Universidade Federal de Minas Gerais Belo Horizonte MG Brasil Hospital das Clínicas da Universidade Federal de Minas Gerais,Belo Horizonte, MG – Brasil; 3 Fundação Centro de Hematologia e Hemoterapia de Minas Gerais Belo Horizonte MG Brasil Fundação Centro de Hematologia e Hemoterapia de Minas Gerais (HEMOMINAS Foundation), Belo Horizonte, MG – Brasil; 4 Núcleo de Ações e Pesquisa em Apoio Diagnóstico Faculdade de Medicina Universidade Federal de Minas Gerais Belo Horizonte MG Brasil Núcleo de Ações e Pesquisa em Apoio Diagnóstico (Nupad) – Faculdade de Medicina, Universidade Federal de Minas Gerais, Belo Horizonte, MG – Brasil

**Keywords:** Anemia Falciforme, Vasculite, Hemólise, Oclusão Vascular, Hipertensão, Prognóstico, Teste de Esforço, Exercício

## Abstract

**Fundamento:**

Pacientes com anemia falciforme (AF) têm risco aumentado de complicações cardiovasculares. O teste ergométrico é usado como marcador de prognóstico em uma série de doenças cardiovasculares. Entretanto, há uma escassez de evidências sobre exercícios em pacientes com AF, especialmente em relação à sua segurança, viabilidade e possível função prognóstica.

**Objetivos:**

Usamos o teste em esteira máximo para determinar a segurança e a viabilidade do teste ergométrico em pacientes com AF. Além disso, os fatores associados à duração do exercício, bem como o impacto das alterações causadas pelo exercício em resultados clínicos, também foram avaliados.

**Métodos:**

113 pacientes com AF que passaram pelo teste ergométrico e por uma avaliação cardiovascular abrangente incluindo um ecocardiograma e os níveis do peptídeo natriurético do tipo B (BNP). O desfecho de longo prazo foi uma combinação de eventos incluindo morte, crises álgicas graves, síndrome torácica aguda ou internações hospitalares por outras complicações associadas â doença falciforme. A análise de regressão de Cox foi realizada para identificar as variáveis associadas ao resultado. Um p valor <0,05 foi considerado estatisticamente significativo.

**Resultados:**

A média de idade foi de 36 ± 12 anos (intervalo, 18-65 anos), e 62 pacientes eram do sexo feminino (52%). A presença de alterações isquêmicas ao esforço e resposta pressórica anormal ao exercício foram detectadas em 17% e 9 % da´população estudada respectivamente. Dois pacientes apresentaram crise álgica com necessidade de internação hospitalar no período de 48 horas da realização do exame. Fatores associados à duração do exercício foram idade, sexo, velocidade máxima de regurgitação tricúspide (RT), e relação E/e’, após a padronização quanto aos marcadores da gravidade da doença. Durante o período médio de acompanhamento de 10,1 meses (variando de 1,2 a 26), 27 pacientes (23%) apresentaram desfechos clínicos adversos. Preditores independentes de eventos adversos foram a concentração de hemoglobina, velocidade do fluxo transmitral tardio (onda A), e a resposta da PA ao exercício.

**Conclusões:**

A realização de testes ergométricos em pacientes com AF, clinicamente estáveis, é viável. A duração do exercício estava associada à função diastólica e a pressão arterial pulmonar. A resposta anormal da PA foi um preditor independente de eventos adversos.

## Introdução

A anemia falciforme (AF) é um problema de saúde global crescente associado a complicações potencialmente fatais e lesões progressivas dos órgãos.^1 - 4^ Embora se espere que o número de pacientes com AF aumente com a melhoria do tratamento, a expectativa de vida é reduzida em cerca de 3 décadas, mesmo com o melhor atendimento de saúde possível.^1^ Essa doença é caracterizada pela presença de eritrócitos anormais deteriorados pela hemoglobina S, levando a uma disfunção multissistêmica.^2 , 5^ A característica fisiopatológica da AF é a polimerização da hemoglobina que causa vaso oclusão tecidual com lesão de isquemia e reperfusão e pela presença de hemólise.5,6 As complicações crônicas resultam de dois mecanismos principais, incluindo uma vasculopatia de grandes vasos e lesões isquêmicas progressivas aos órgãos.^1 , 2 , 5^

Nas últimas décadas, o diagnóstico precoce e a melhora no tratamento clínico de modo geral prolongaram significativamente a sobrevida de pacientes com AF^7^ e, dessa forma, a detecção das complicações cardiovasculares aumentou. A anemia crônica está associada a várias alterações cardíacas bem descritas em pacientes com AF, incluindo a dilatação do ventrículo esquerdo, aumento da massa, e função diastólica prejudicada.^8 - 10^ Além disso, a hemólise intravascular pode levar à hipertensão pulmonar pré-capilar, que é uma das principais complicações da AF, com consequências graves para as câmaras direitas do coração.^3 , 11 - 17^

Pacientes com AF têm risco aumentado de isquemia miocárdica e morte súbita, especialmente com o envelhecimento da população afetada.^6 , 11 , 18^ A dor no peito geralmente é atribuída à crise vaso-oclusiva e o diagnóstico de infarto do miocárdio geralmente é ignorado, ocasionalmente feito na autópsia.^18^ Portanto, a doença cardíaca isquêmica pode estar presente em um número significativo de pacientes com AF.

O teste ergométrico é amplamente utilizado na pesquisa de isquemia miocárdica em pacientes com dor torácica ou com possíveis sintomas equivalentes isquêmicos.^19^ Sua utilização em pacientes com AF, entretanto, pode levar a alterações metabólicas induzidas pelo exercício que poderiam favorecer o afoiçamento dos eritrócitos e promover oclusões vasculares.^20 , 21^ Esse fato suscitou um dilema entre recomendar exercícios para esses pacientes ou privá-los dos efeitos benéficos da atividade física. Embora estudos anteriores tenham demonstrado uma tolerância normal ao exercício em pacientes com AF,^22 , 23^ eles tinham várias limitações, incluindo um pequeno número de pacientes e o uso de testes de caminhada de seis minutos para avaliar a capacidade funcional. Portanto, há escassez de evidências que indiquem programas de exercícios para pacientes com AF. Além disso, não está claro se os parâmetros obtidos em pacientes submetidos a teste de esforço máximo limitado por sintomas poderiam apresentar associação com presença de eventos adversos no seguimento dos pacientes com AF

Portanto, este estudo buscou 1) verificar a tolerância ao exercício em pacientes com AF; 2) determinar os fatores associados à duração do teste ergométrico; 3) examinar o impacto e os parâmetros da resposta cardiovascular induzidos pelo esforço na ocorrência de desfechos clínicos; 4) avaliar a viabilidade e a segurança dos testes ergométricos na população com AF.

## Métodos

### População do estudo

Este foi um estudo de centro único em que foram cadastrados pacientes com AF confirmada por eletroforese de hemoglobina. Foram excluídos pacientes que não conseguiram realizar o teste ergométrico devido a problemas ortopédicos ou a outros problemas orgânicos graves associados à AF (crise álgica aguda, insuficiência venosa grave, descompensação cardiovascular ou respiratória).

Os níveis de peptídeo natriurético tipo B (BNP) foram medidos utilizando-se radioimunoensaio padrão em todos os pacientes imediatamente antes do teste ergométrico. O protocolo de pesquisa foi aprovado pelo Comitê de Ética da Universidade Federal de Minas Gerais, e o consentimento informado por escrito foi obtido de todos os pacientes.

### Protocolo do teste ergométrico

Foi realizado um exercício limitado por sintomas em uma esteira (Centurium 200, Micromed, Brasil), utilizando o protocolo de Bruce modificado, que apresenta, nos estágios iniciais, incrementos menores da carga de esforço permitindo melhor adaptação e tolerância ao exercício. Esse protocolo é derivado do protocolo padrão de Bruce e apresenta estágios de 3 minutos, sendo diferente apenas no primeiro, que apresenta velocidade inicial usual do primeiro estágio do protocolo original e altera apenas a inclinação (sem inclinação nos primeiros 3 minutos). O segundo estágio é semelhante ao primeiro estágio original de Bruce, e, depois disso, o protocolo usual é seguido. Dessa forma, a relação entre esforço e consumo de O_2_ é de cerca de 0,5 MET/minuto até o terceiro estágio, e, daí em diante, ± 1,2 MET/minute.^19^

Um ECG de 13 derivações foi monitorado continuamente e registrado a cada minuto. A pressão arterial foi registrada com manguito em repouso, durante os últimos 30 segundos de cada estágio, e durante o período de recuperação de 6 minutos. Depois de atingir o esforço máximo, todos os pacientes passaram 1 minuto em período de desaceleração gradual, recuperação ativa, a uma velocidade de 2,4 km por hora e uma inclinação de 2,5 por cento. Depois de 1 minuto, todos os pacientes concluíram a fase de recuperação na posição supina.

O teste foi máximo, com os pacientes permanecendo na esteira até atingirem parâmetros subjetivos (dispneia, fadiga, dor no peito ou membros inferiores, incapacidade de continuar na esteira) de intolerância ou exercício, ou contraindicações usuais para a continuidade do exercício (como arritmias persistentes). O VO_2_ de pico e os MET foram estimados no pico do exercício. Foram avaliadas a presença de alterações patológicas do segmento ST-T; a resposta cronotrópica e pressórica ao esforço e a ocorrência de arritmias cardíacas. A resposta anormal da pressão arterial ao exercício foi definida como ausência de elevação ou aumento da pressão arterial sistólica no pico do exercício <20 mmHg, ou uma queda da pressão arterial sistólica abaixo do valor em repouso durante o exercício.^24^ Alterações no segmento ST foram consideradas indicativas de isquemia quando houve uma depressão do segmento ST horizontal ou com inclinação para baixo ≥ 1 mm a 60–80 ms após o ponto J.^19^

Foi realizada a oximetria em repouso e durante o teste ergométrico utilizando-se dois oxímetros: OHMEDA 3800, GE e HELLCOR OXIMAX N-600X, um em cada dedo indicador. Todos os exames foram realizados e analisados por um cardiologista experiente.

### Avaliação ecocardiográfica

O ecocardiograma foi realizado de acordo com as recomendações da *American Society of Echocardiography* (Sociedade Americana de Ecocardiografia)^25^ utilizando-se um ecocardiograma comercialmente disponível (GE Vivid Q, Horten, Noruega). A fração de ejeção do VE foi calculada conforme a regra de Simpson modificada, e a massa do VE foi calculada utilizando-se a fórmula de Devereux.^26^ A função diastólica foi avaliada por exame de Doppler de onda de pulso do influxo mitral, e por imagem de Doppler tecidual.^27^ A velocidade diastólica precoce (e’) na borda medial do anel mitral foi obtida, e a relação entre o pico mitral E e a velocidade e’ (Relação E/e’) foi calculada. A função ventricular direita foi avaliada utilizando-se a velocidade sistólica de pico no anel tricúspide utilizando-se imagens de Doppler tecidual,^28^ o movimento anular tricúspide e a alteração da área fracionada, que foi calculada como (área diastólica final do VD – área sistólica final do VD)/área diastólica final do VD x 100. A velocidade máxima de regurgitação tricúspide (RT) foi obtida nas vistas de 4 câmaras ou paraesternal. Todas as medições foram realizadas por um único investigador, de forma cega quanto aos dados clínicos, e a média foi de 3 batimentos.

### Definições de desfecho

O desfecho principal foi a duração do exercício e o desfecho secundário foi uma combinação dos seguintes eventos: (1) morte relacionada a AF, (2) mortalidade global, (3) três ou mais episódios de dor aguda que exigem internação hospitalar, (4) síndrome aguda do tórax caracterizada por um infiltrado pulmonar recente detectado por radiografia do tórax associada a dor no peito, febre, taquipneia, chiado, tosse e hipoxemia, (5) hospitalização por outras complicações relacionadas à AF, especialmente infecção potencialmente fatal.

A data do cadastro no estudo foi definida como sendo a data em que o teste ergométrico foi realizado. O período de inclusão foi de agosto de 2015 a setembro de 2016, e o acompanhamento terminou em novembro de 2017. Os dados do acompanhamento foram obtidos durante consultas clínicas de acompanhamento ou entrevistas telefônicas.

### Análise estatística

O estudo foi projetado para alcançar 90% de poder de detecção de uma prevalência de 15% de anormalidades de ECG sugeridas de isquemia miocárdica na população geral com AF. Consideramos que pelo menos 10 pacientes terão anormalidades ST-T isquêmicas, retornando um tamanho de amostra estimado de 93.

Dados categóricos foram apresentados como números e porcentagens, e dados contínuos foram expressos como média ± desvio padrão (DP) ou mediana e faixa interquartil, dependendo do padrão de distribuição de cada variável. O teste de Shapiro-Wilk foi realizado para avaliar a distribuição das variáveis contínuas.

Para se determinarem os fatores associados à duração do teste ergométrico, foram realizados modelos de regressão linear com análise univariada e multivariada. As premissas da análise de regressão linear foram verificadas sem que se observassem quebras significativas.

A análise de regressão de Cox foi realizada para determinar as características que estavam associadas de maneira independente à combinação de desfechos. Variáveis de testes clínicos, laboratoriais, ecocardiográficos e da ergometria que eram clinicamente relevantes e significativamente associados a eventos em análise univariadas foram incluídas no modelo de regressão logística multivariável. As variáveis que entraram no modelo final foram parâmetros de idade, sexo, testes laboratoriais (concentrações de reticulócitos e de hemoglobina), ecocardiográficos (velocidade máxima de RT, relação E/e’, e massa do VE indexada), e ergométrico (resposta à pressão anormal, e presença de isquemia). Um p valor <0,05 foi considerado estatisticamente significativo.

A análise estatística foi realizada utilizando-se o software SPSS, versão 22.0 (SPSS Inc., Chicago, Illinois, EUA).

## Resultados

### Características clínicas da população do estudo

Um total de 120 pacientes ambulatoriais foram incluídos, porém 7 deles não conseguiram fazer o exame na sala de exercícios, resultando em um total de 113 pacientes que concluíram o protocolo de estudo. Destes, 71 eram portadores de hemoglobina (Hb) SS, 40 de HbSC, e 2 eram portadores de anemia falciforme-talassemia beta zero (Hb S-β^0^-thal). A média de idade dos pacientes foi de 36,2 ± 12,4 anos (intervalo, 18-65 anos), e 62 pacientes eram do sexo feminino (52%). A maioria dos pacientes era assintomática, estava dentro da classe funcional (CF) NYHA I (77%), enquanto 24 (20%) estavam dentro da classe II, e 4 (3%), na classe III. As características clínicas da população do estudo estão resumidas na Tabela 1 . Dezesseis pacientes (13%) tinham hipertensão e 43 pacientes (36%) tinham disfunção renal. A internação hospitalar no ano anterior havia ocorrido em 25 pacientes (21%), e 2 ou mais vezes em 11 pacientes (9%).

**Tabela 1:** – Características de linha de base da população do estudo

Variáveis*	Valor
Área da superfície corporal (m^2^)	1,7 ± 0,2
Frequência Cardíaca (bpm)	75,8 ± 13,6
Pressão arterial sistólica/diastólica (mmHg)	117,4 ± 14,6/73,2 ± 4,3
Hemoglobina (g/dL)	9,9 ± 2,2
Reticulócitos (% de eritrócitos)	5,6 [3,6/8,7]
Contagem de leucócitos (x10^3^/l)	8,6 ± 3,0
Desidrogenase láctica (U/l)	575 [413/833]
Aspartato aminotransferase (U/l)	23 [16/32]
Ferritina (ng/ml)	181 [75/388]
Bilirrubina total (mg/dl)	1,7 [1,1/3,0]
Creatinina (mg/dl)	0,7 [0,6/0,8]
Peptídeo natriurético tipo B (BNP, pg/ml)	27 [11/62]
**Medições ecocardiográficas**	
Diâmetro diastólico final do ventrículo esquerdo (mm)	51 [48/56]
Diâmetro sistólico final do ventrículo esquerdo (mm)	33 [30/37]
Fração de ejeção ventricular esquerda (%)	63 [59/65]
Massa do VE indexada (g/m^2^)	103,2 [85/130]
Pico de velocidade diastólica precoce do fluxo transmitral (E,cm/s)	93,1 ± 22,2
Pico de velocidade tardia do fluxo transmitral (A, cm/s)	55,9 ± 16,9
Tempo de desaceleração (ms)	206,0 ± 41,6
Relação E/e’	1,8 ± 0,7
Relação E/e’ †	6,8 ± 2,2
Volume atrial esquerdo indexado (mL/m^2^)	42,1 ± 14,8
Alteração de área fracionada do ventrículo direito (%)	44,2 ± 5,8
Pico de velocidade sistólica do ventrículo direito (cm/s)	14,5 ± 3,0
Movimento anular tricúspide (mm)	25,9 ± 4,3
Índice de desempenho miocárdico do ventrículo direito	0,12 ± 0,07
Velocidade máxima de regurgitação tricúspide‡ (m/s)	2,2 ± 0,3
Área do átrio direito (cm^2^)	16,2 ± 3,4

*Valores expressos com valor médio ± DP, ou mediana [faixa interquartil]. e’: velocidade diastólica precoce do anel mitral nos anéis mitrais do septo e lateral, E/A: relação entre velocidade do fluxo transmitral precoce e tardia. † E/e’: Relação entre velocidade diastólica precoce do fluxo transmitral e velocidade diastólica precoce do anel mitral (média nos anéis mitrais do septo e lateral). ‡Pico de velocidade sistólica no anel tricúspide por imagem Doppler tecidual.

Acidentes vasculares cerebrais foram diagnosticados anteriormente em 16 pacientes (13%) que estavam sob regime de hipertransfusão e não apresentavam sequelas motoras significativas. Os medicamentos usados mais frequentemente foram o ácido fólico (93%), a hidroxiureia (62%), e os inibidores de enzima conversora da angiotensina ou bloqueadores de receptores de angiotensina (23%). Sete pacientes (6%) estavam tomando furosemida. Todos os pacientes que estavam clinicamente estáveis apresentaram anemia leve, com níveis de hemoglobina de 9,9 ± 2,2 g/dl ( Tabela 1 ). As concentrações de peptídeo natriurético tipo B estavam dentro da faixa normal.

As medições ecocardiográficas são apresentadas na Tabela 1 . A maioria dos pacientes tinha dimensões ventriculares normais com função sistólica preservada. O volume atrial esquerdo estava aumentado, enquanto outros parâmetros para avaliar a função diastólica estavam normais, especialmente a relação E/e’ derivada de Doppler tecidual, que estava dentro do intervalo normal. Da mesma forma, as dimensões do ventrículo direito e o jato de velocidade máxima de regurgitação tricúspide também estavam dentro do intervalo normal. Apenas 2 pacientes tinham jato de velocidade de regurgitação tricúspide ≥3 m/s.

### Teste ergométrico

Foram detectadas anormalidades de ST isquêmicas compatíveis com os critérios de isquemia durante o esforço em 19 pacientes (17%). As características do teste ergométrico são apresentadas na Tabela 2 .

**Tabela 2:** – Características do paciente durante o teste ergométrico

Variáveis*	Valor
Oximetria (%)	95 [92/96]
Pico de FC (batimentos/minuto)	158,4 ± 21,0
Pico de FC (% prevista)	86,9 ± 10,0
Pico de VO_2_ (ml.Kg^-1^.min^-1^)	31,0 ± 9,7
MET	8,9 ± 2,8
Presença de isquemia	20 (17)
Batimentos supraventriculares precoces	40 (33)
Batimentos ventriculares precoces	17 (14)
Resposta de pressão arterial anormal	11 (9)
Alterações na pressão arterial sistólica (mmHg) †	27,5 ± 14,9
Delta da pressão sistólica/duração do exercício (mmHg/min) ‡	3,1 ± 1,5

*Valores expressos com valor médio ± DP, número (porcentagem) de pacientes, ou mediana [faixa interquartil]. † Pressão arterial sistólica no pico - em repouso. † Pressão arterial sistólica no pico - em repouso/ tempo de exercício; FC: frequência cardíaca; MET: Metabolic equivalente of task, ou, equivalente metabólico de uma tarefa; sendo que um MET é definido como a quantidade de oxigênio consumido enquanto um ser humano está sentando, em repouso, sendo igual a 3.5 ml O_2_ por kg x min. O conceito de MET representa um modelo prático para expressar o custo energético de diversas atividades e para estimar o condicionamento físico.

**Figura 1:**
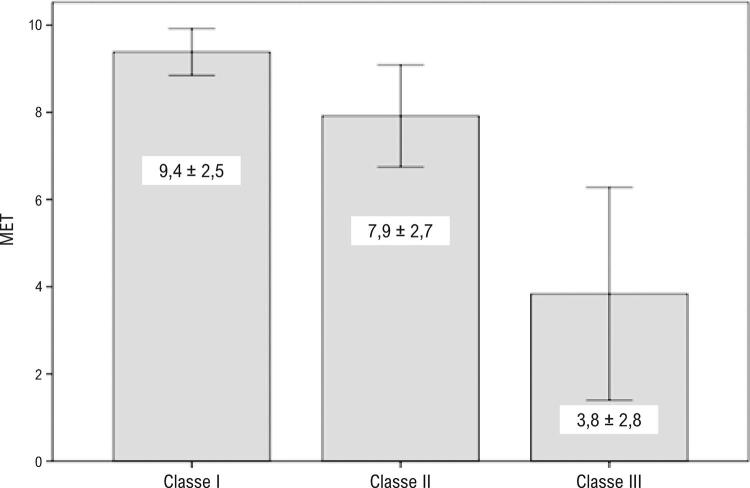
– Associação entre capacidade funcional por classe funcional NYHA avaliada por anamnese, e capacidade de exercício medida por ergometria.

Na população em geral, a avaliação subjetiva da capacidade funcional durante a anamnese pela classe funcional (CF) NYHA estava associada à medida durante o teste ergométrico. A capacidade funcional foi medida em MET, com o valor médio de 8,9 ± 2,8, no intervalo entre 1,5 e 17,3. Os pacientes de classe I alcançaram 9,4 MET, enquanto os de classe III alcançaram menos de 4 MET. A relação entre classe funcional, conforme avaliada por anamnese e ergometria é apresentada na Figura 1 .

A presença de extrassístoles supraventriculares precoces foi frequente durante o exercício, isoladas em 16% dos casos, e complexas com alguns episódios de taquicardia paroxística supraventricular em 17% dos pacientes. Extrassístoles ventriculares precoces isoladas ocorreram em 14 pacientes (12%). Foi detectada resposta anormal da pressão arterial em 10 pacientes (9%), com o aumento médio da pressão arterial sistólica de 14 mmHg em comparação com os que tem resposta normal, nos quais o aumento médio da pressão arterial foi 29 mmHg (p=0,002). Após o teste ergométrico, no período de 48 hours, dois pacientes (1,8%) passaram por crises de dor que exigiram a internação hospitalar para tratamento.

### Fatores associados à duração do exercício

Na população geral, a duração do exercício foi de 9,2 minutos, no intervalo de 1,1 a 15,5 minutos. Várias variáveis clínicas, laboratoriais e ecocardiográficas foram testadas quanto a uma possível associação à tolerância ao exercício ( Tabela 3 ). Os possíveis preditores que foram selecionados para o modelo multivariado foram idade, sexo, oximetria em repouso, concentração de hemoglobina, e parâmetros ecocardiográficos de função diastólica de VE, função de VD e pressão pulmonar avaliada pela velocidade máxima de RT. A velocidade máxima de RT e a relação E/e’ foram os principais fatores associados ao tempo de exercício na análise univariada. Na análise de regressão linear multivariada incluindo os marcadores laboratoriais da gravidade da doença, a velocidade máxima de RT e a relação E/e’ surgiram como fatores importantes associados à duração do exercício, após a padronização quanto a idade e sexo ( Tabela 4 ).

**Tabela 3:** – Fatores associados à duração do exercício

Variáveis	Univariada		Multivariada	

	Beta	p-valor	Beta	p-valor
Idade (anos)	-0,067	0,001	-0,038	0,045
Sexo feminino	1,386	0,003	1,195	0,006
Betabloqueadores	-2,158	0,014	…	…
Úlceras na perna	-1,242	0,034	…	…
Acidente vascular cerebral anterior	-1,475	0,042	…	…
Volume de AE indexado (mL/m^2^)	-0,049	0,002	…	…
Pico de velocidade A (cm/s)	-0,032	0,025	…	…
Tempo de desaceleração (ms)	-0,017	0,004	…	…
Relação E/e’	-0,358	<0,001	-0,224	0,018
Velocidade máxima de RT (m/s)	-2,675	<0,001	-1,810	0,015
Massa do VE indexada (g/m^2^)	-0,015	0,014	…	…
Pressão arterial sistólica (mmHg)	-0,045	0,005	…	…
Oximetria (%) em repouso	0,240	0,022	…	…
Peptídeo natriurético tipo B (pg/ml)	-0,006	0,001	…	…
Hemoglobina (g/dl)	0,387	<0,001	…	…
Ferritina (ng/ml)	-0,001	0,001	…	…
Desidrogenase láctica (IU/l)	-0,002	0,004	…	…
Proteinúria	1,436	0,005	…	…

*AE: átrio esquerdo; VE: ventrículo esquerdo; RT: regurgitação tricúspide.*

**Tabela 4:** – Análise de risco proporcional de Cox para previsão de resultados adversos em pacientes com anemia falciforme

Variáveis	Univariada		Multivariada	

	FC (IC 95%)	p-valor	FC (IC 95%)	p-valor
Genótipo Hb SS	2,546 (1,020-6,351)	0,045	…	…
Hemoglobina (g/dl)	0,803 (0,664-0,970)	0,023	0,688 (0,552-0,858)	0,001
Massa de VE (g/m^2^)	1,007 (1,000-1,015)	0,055	…	…
VAE (mL/m^2^)	1,022 (0,999-1,046)	0,060	…	…
Área do átrio direito (cm^2^)	1,143 (1,042-1,255)	0,005	…	…
TAM (mm)	1,098 (1,008-1,197)	0,033	…	…
Velocidade de RT (m/s)	3,729 (1,474-9,433)	0,005	…	…
Pico de velocidade A (cm/s)	0,976 (0,955-0,998)	0,031	0,964 (0,933-0,997)	0,034
Resposta anormal de PAS	4,110 (1,346-12,550)	0,013	4,990 (1,316-18,921)	0,018
BNP (pg/ml)	1,001 (1,000-1,003)	0,052	…	…

*IC: intervalo de confiança; RC: razão de chance; VAE: volume do átrio esquerdo; VE: ventrículo esquerdo; PAS: pressão arterial sistólica; TAM: movimento anular tricúspide; RT: regurgitação tricúspide; BNP: peptídeo natriurético tipo B.*

### Preditor de eventos adversos.

Durante um período de acompanhamento médio de 10,1 meses (intervalo de 1,2 a 26), 27 pacientes (23%) apresentaram desfechos clínicos desfavoráveis: 4 pacientes morreram (uma morte não estava relacionada à AF), 8 foram hospitalizados devido a >= 3 episódios de crise álgica aguda, 11 tiveram síndrome torácica aguda e 4 foram hospitalizados com outras complicações relacionadas à AF.

**Figura 2:**
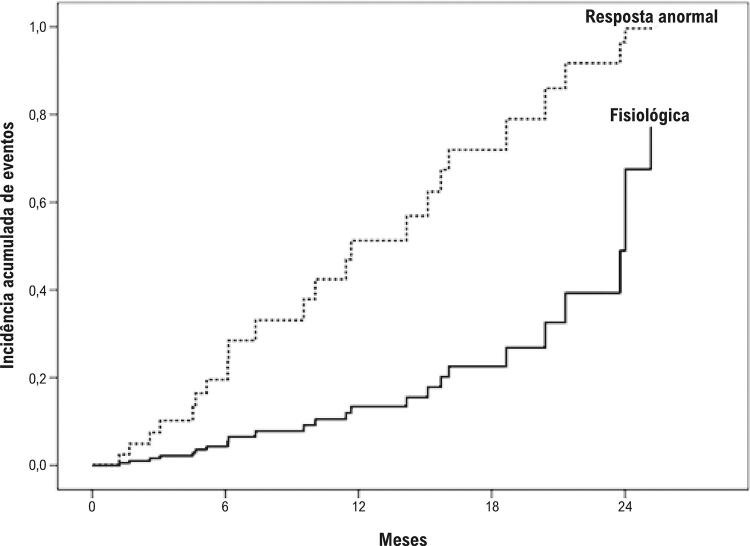
– Incidência acumulada de eventos adversos em pacientes com AF que apresentaram resposta anormal da pressão arterial ao exercício comparados àqueles com resposta fisiológica (p-valor de 0,027).

Várias variáveis foram testadas quanto a uma possível associação a resultados adversos ( Tabela 4 ). Os possíveis preditores que foram selecionados para o modelo multivariado foram o genótipo Hb SS, níveis de hemoglobina, massa do ventrículo esquerdo, volume do átrio esquerdo, área do átrio direito, velocidade de pico da regurgitação tricúspide, velocidade de pico A transmitral, níveis de BNP, e resposta de pressão arterial anormal ao exercício. Na análise multivariada, os preditores independentes de eventos adversos foram a concentração de hemoglobina, velocidade de pico A transmitral, e a resposta da pressão arterial ao exercício. A incidência acumulada de eventos adversos pela resposta da pressão arterial sistólica é apresentada na Figura 2 .

## Discussão

Este estudo busca apresentar informações sobre a tolerância a exercícios em pacientes com AF. Como existe uma falta de evidências na literatura sobre teste ergométrico na AF, nossos resultados mostram que o teste ergométrico em pacientes crônicos com AF compensada é viável, relativamente seguro e pode ser realizado em ambiente hospitalar com uma equipe experiente. Além disso, o teste ergométrico oferece informações úteis para o controle de pacientes com AF.

Há escassez de evidências que indiquem um programa de exercícios para pacientes com AF. A principal questão enfrentada por profissionais de saúde envolvidos no controle de AF é o nível seguro de exercícios físicos que devem recomendar a seus pacientes.^21^

Como é sabido que a atividade física induz alterações metabólicas que podem precipitar crises vaso-oclusivas, os pacientes geralmente são incentivados a praticar exercícios limitados pelo aparecimento de sintomas. A presença da anemia induz a uma transição mais rápida do metabolismo aeróbico ao anaeróbio durante o exercício, o que pode estimular a polimerização da hemoglobina S e causar oclusões microvasculares.^29 , 30^ Além disso, a desidratação que ocorre durante o exercício, associada aos episódios agudo de hipóxia tecidual, também pode contribuir para o afoiçamento das hemácias. Portanto, embora nosso estudo e outros tenham demonstrado a segurança relativa da atividade física em pacientes com AF,^21^ ela ainda representa alguns riscos. Observamos duas complicações após o teste, o que reforça a necessidade de cuidados médicos, incluindo hidratação, para a realização de testes ergométricos nessa população vulnerável.

Entretanto, evidências recentes sugerem que pacientes com AF podem fazer atividades físicas, ainda que sejam necessárias recomendações específicas sobre a duração e a intensidade do exercício.^21 , 30^

A presença de arritmias durante o exercício varia muito na literatura. Em nosso estudo, 16% dos pacientes apresentaram arritmias supraventriculares, um número mais alto do que o esperado para esse grupo de pacientes.^19 , 31^ Isso provavelmente se deve ao aumento atrial e à disfunção diastólica geralmente observada com a AF, que são os principais fatores associados a essas arritmias,^32^ padronizado por idade. A prevalência de arritmias ventriculares foi semelhante aos dados da literatura.^19^ A presença de alterações isquêmicas do segmento ST, sugerindo isquemia miocárdica, é considerada frequente na AF, variando entre 10% e 50%,^6 , 11 , 33^ Encontramos uma prevalência de 17%, sem que houvesse quaisquer outros achados indicando doença coronária obstrutiva.

### Determinantes da tolerância ao exercício em pacientes com AF

Pacientes com AF apresentam, consistentemente, deficiência na capacidade de fazer exercícios. Vários fatores contribuem para a intolerância ao exercício, incluindo possíveis anormalidades no enchimento cardíaco, anemia crônica, doença vascular pulmonar, e doença vascular periférica relacionada à oclusão microvascular.^11 , 21 , 34 , 35^ Foram propostos três mecanismos principais de limitação do exercício na AF: anemia, doença vascular pulmonar, e doença vascular periférica e/ou miopatia.^21^ Realmente, em nosso estudo, a velocidade da regurgitação tricúspide que estima a pressão sistólica da artéria pulmonar se manteve como determinante importante da duração do exercício, após a padronização por idade e sexo. Da mesma forma, a relação E/e’ derivada de Doppler tecidual, que é um marcador da pressão de enchimento do VE alta, foi um fator independente associado à duração do exercício.

Em conformidade com nossos achados, um estudo anterior mostrou que uma redução na distância da caminhada de 6 minutos estava independentemente associada a medições ecocardiográficas de hipertensão pulmonar, expressa pela velocidade da regurgitação tricúspide, e a medidas de disfunção diastólica, sugerindo duas determinantes independentes de intolerância ao exercício.^36^

Na população geral, anormalidades na função diastólica do ventrículo esquerdo, medidas pela relação E/e’, são independentemente associadas à capacidade de exercício.^37^ Embora os pacientes do sexo masculino tivessem uma capacidade de exercício maior que a dos pacientes do sexo feminino, a magnitude de sua diferença diminuía com a idade. Comparados aos indivíduos com função diastólica normal, os pacientes com disfunção diastólica leve (relaxamento deficiente) tinham um aumento progressivo na magnitude da capacidade do exercício com o avanço da idade.^37^ No presente estudo, com pacientes assintomáticos com disfunção diastólica leve, a idade estava inversamente relacionada à capacidade de exercício.

### Resposta de pressão arterial anormal e resultado adverso na AF

A pressão arterial média deve normalmente aumentar em cerda de 40% com o exercício incremental, devido ao aumento do débito cardíaco, com um aumento progressivo da pressão arterial sistólica.^24^ Respostas de pressão arterial anormais são relativamente comuns e seu potencial de valor clínico tem chamado cada vez mais atenção.^38^ Embora seja difícil determinar a prevalência exata de resposta pressórica anormal ao esforço, devido a variações na definição desse parâmetro, estima-se em até 6% a ocorrência dessa alteração ao esforço.

A hipotensão induzida pelo exercício tem sido considerada, há muito tempo, um sinal de prognóstico ruim para os indivíduos portadores de doença cardiovascular estabelecida.^40 - 42^ Uma análise sistemática e uma meta-análise demonstraram que a resposta hipotensiva prevê eventos cardiovasculares fatais e não fatais de longo prazo e mortalidade global.^43^ Isso foi observado independentemente da apresentação da doença, do modo de exercício realizado, da intensidade do exercício, ou de como a hipotensão pelo exercício era definida. Em conformidade, identificamos que a resposta pressórica anormal foi um preditor independente de eventos adversos, após o ajuste para fatores prognósticos conhecidos.

Vários mecanismos foram propostos para explicar a associação entre o aumento no risco da ocorrência de eventos cardiovasculares adversos e a presença de resposta pressórica anormal (elevação insuficiente ou queda da PA) durante o teste ergométrico.^38 , 42^ Durante o exercício, a pressão arterial sistólica abaixo dos valores de repouso foi associada a doença cardiovascular subjacente, incluindo disfunção do ventrículo esquerdo, doença arterial coronariana, e obstruções do fluxo aórtico.^42 , 43^ Anormalidades no sistema nervoso autônomo durante o teste ergométrico provavelmente são observadas em pacientes que apresentaram respostas de pressão arterial sistólica diminuídas. O desequilíbrio autonômico está relacionado ao desenvolvimento de insuficiência cardíaca, e distúrbios similares possivelmente ocorrem nas pessoas com resposta de pressão arterial sistólica ao exercício diminuída.^44^ Um estudo anterior demonstrou que até mesmos as elevações modestas da pressão arterial sistólica durante o teste ergométrico de esforço estão associadas a um risco menor de mortalidade global e infarto do miocárdio.^42^ Entretanto, a etiologia da hipotensão induzida pelo exercício é multifatorial e complexa.

Em pacientes com AF é descrito que a pressão arterial sistêmica é mais baixa nos pacientes com AF sem comorbidades quando comparado à população em geral.^45^ Pacientes com AF cujos valores de pressão arterial estão acima da faixa esperada para esse população – “hipertensão sistêmica relativa” – tinham risco maior de acidente vascular cerebral e morte.^46^ O mecanismo exato pelo qual a resposta anormal da pressão arterial induzida pelo exercício em pacientes com AF está relacionada a resultados adversos precisa ser definido. A isquemia miocárdica induzida pelo exercício pode causar disfunção do ventrículo esquerdo. Realmente, um estudo anterior relatou que o volume diastólico final do ventrículo esquerdo diminuiu mais significativamente com o exercício em pacientes que apresentaram ECG isquêmico.^47^ É descrito também que pacientes com a presença de resposta isquêmica ao esforço também atingiram um duplo produto (pressão arterial sistólica x FC) mais alto, com uma elevação excessiva da pressão arterial, sugerindo aumento da demanda de oxigênio do miocárdio durante o exercício na população.^48^

A presença de hipertensão pulmonar está associada à limitação do exercício e prognóstico ruim em pacientes com AF.^21^ Embora, em nosso estudo, a resposta da pressão pulmonar ao exercício não tenha sido avaliada, sua elevação excessiva durante o exercício pode contribuir para a disfunção do ventrículo direito, e a redução do débito cardíaco, com a consequente resposta hipotensiva ao exercício. Diante de exposto, a relação entre a ocorrência de eventos clínicos adversos e a resposta pressórica anormal ao esforço em pacientes com AF é complexa e provavelmente mediada por complicações crônicas, incluindo anemia, doença vascular pulmonar, e disfunção diastólica do ventrículo esquerdo.

### Limitações do estudo

Este estudo tem algumas limitações. O tamanho da amostra foi estimado para se detectar anormalidades no ECG relacionadas a isquemia miocárdica na AF, o que limita a análise em termos dos preditores de eventos adversos. Os pacientes cadastrados neste estudo são encaminhados de atendimento ambulatorial, incluindo um amplo espectro de pacientes com AF, mas com um pequeno número de pacientes de subgrupos mais graves, especialmente aqueles com hipertensão pulmonar, o que limita a validade externa para pacientes mais graves.

34% dos pacientes eram portadores do subtipo SC, em geral menos graves, o que limita nossas conclusões para toda a população com doença falciforme (subtipo SS e outros). Outros dois fatores limitantes foram a impossibilidade de se utilizar o teste de esforço cardiopulmonar, ferramenta ideal, mas menos disponível, para avaliação da capacidade funcional nesses pacientes, e a dificuldade na mensuração da pressão arterial durante o esforço, já descrita em outros estudos que utilizaram a ergometria, e que pode comprometer a reprodutibilidade dos nossos achados.

## Conclusões

A realização de testes ergométricos em pacientes com AF clinicamente estáveis é relativamente segura e viável, e oferece informações clínicas valiosas, além de poder ser útil na avaliação do condicionamento aeróbico. Alterações eletrocardiográficas isquêmicas induzidas pelo exercício foram frequentes e a ocorrência de crises álgicas após o esforço não foi comum. Os principais determinantes da duração do exercício foram a função diastólica do ventrículo esquerdo e a pressão arterial pulmonar estimada pela velocidade da regurgitação tricúspide. A resposta pressórica anormal ao esforço foi um preditor independente de eventos adversos. São necessários estudos adicionais para se determinar a segurança do exame em amostras maiores assim como para esclarecer os mecanismos subjacentes associados ao risco aumentado de eventos adversos em pacientes com AF com resposta de pressão arterial sistólica diminuída durante o teste ergométrico.
