# Supplementary material for: Testes Ergométricos em Pacientes com Anemia Falciforme: Segurança, Viabilidade e Possíveis Implicações no Prognóstico
Source: Arq Bras Cardiol. 2022 Mar 10;118(3):565–75. [Article in Portuguese] doi: 10.36660/abc.20200437 (PMC8959037; doi:10.36660/abc.20200437)
Supplement: Translation

# Translation: Exercise Testing In Patients with Sickle Cell Disease: Safety, Feasibility and Potential Prognostic Implication

**Authors:** Christiano Gonçalves de Araújo, Maria Betânia Solis Resende, Julia Teixeira Tupinambás, Rebeca Coeli Teodoro Maciel Dias, Flávio Coelho Barros, Maria Carmen Melo Vasconcelos, José Nelio Januário, Antonio Luiz Pinho Ribeiro, Maria Carmo P. Nunes

**Affiliations:** 1 Brazil Programa de Pós-graduação em Infectologia e Medicina Tropical, Faculdade de Medicina, Universidade Federal de Minas Gerais, Belo Horizonte, MG – Brazil; 2 Brazil Hospital das Clínicas da Universidade Federal de Minas Gerais,Belo Horizonte, MG – Brazil; 3 Brazil Fundação Centro de Hematologia e Hemoterapia de Minas Gerais (HEMOMINAS Foundation), Belo Horizonte, MG – Brazil; 4 Brazil Núcleo de Ações e Pesquisa em Apoio Diagnóstico (Nupad) – Faculdade de Medicina, Universidade Federal de Minas Gerais, Belo Horizonte, MG – Brazil

**Keywords:** Anemia, Sickle Cell, Vasculitis, Hemolysis, Vascular Occlusion, Hypertension, Prognosis, Exercise

## Abstract

**Background:**

Patients with sickle cell disease (SCD) are at increased risk for cardiovascular complications. Exercise testing is used as a prognostic marker in a variety of cardiovascular diseases. However, there is a lack of evidence on exercise in SCD patients, particularly regarding its safety, feasibility, and possible prognostic role.

**Objectives:**

We used the maximal treadmill test to determine safety and feasibility of the exercise testing in SCD patients. Additionally, the factors associated with exercise duration, as well as the impact of exercise-induced changes on clinical outcome, were also assessed.

**Methods:**

One-hundred thirteen patients with SCD, who underwent exercise testing, were prospectively enrolled. A comprehensive cardiovascular evaluation, including echocardiography and B-type natriuretic peptide (BNP) levels, were obtained. The long-term outcome was a composite endpoint of death, severe acute painful episodes, acute chest syndrome, or hospitalization for other SCD-related complications. Cox regression analysis was performed to identify the variables associated with the outcome. A p-value<0.05 was considered to be statistically significant.

**Results:**

The mean age was 36 ± 12 years (range, 18-65 years), and 62 patients were women (52%). Ischemic electrocardiogram and abnormal blood pressure (BP) response to exercise were detected in 17% and 9%, respectively. Two patients experienced pain crises within 48 hours that required hospitalization. Factors associated with exercise duration were age, sex, tricuspid regurgitation (TR) maximal velocity, and E/e’ ratio, after adjustment for markers of disease severity. During the mean follow-up of 10.1 months (ranging from 1.2 to 26), the endpoint was reached in 27 patients (23%). Independent predictors of adverse events were hemoglobin concentration, late transmitral flow velocity (A wave), and BP response to exercise.

**Conclusions:**

Exercise testing in SCD patients who were clinically stable is feasible. Exercise duration was associated with diastolic function and pulmonary artery pressure. Abnormal BP response was an independent predictor of adverse events.

## Introduction

Sickle cell disease (SCD) is an increasing global health problem associated with life-threatening complications and progressive organ damage.^1 - 4^ Although the number of patients with SCD is expected to increase with treatment improvement, life expectancy is reduced by about 3 decades, even with the best medical care.^1^ This condition is characterized by the presence of abnormal erythrocytes damaged by hemoglobin S, leading to a multisystem disorder.^2 , 5^ The pathophysiological hallmark of SCD is hemoglobin polymerization, causing vaso-occlusion with ischemia-reperfusion injury and hemolysis.^5 , 6^ Chronic complications result from two main mechanisms including large-vessel vasculopathy and progressive ischemic organ damage.^1 , 2 , 5^

In recent decades, early diagnosis and effective treatment have greatly prolonged the survival of patients with SCD^7^ and thus cardiovascular complications have been increasingly detected. Chronic anemia is associated with several well-described cardiac changes in patients with SCD, including left ventricular dilation, increased mass, and impaired diastolic function.^8 - 10^ In addition, intravascular hemolysis may lead to precapillary pulmonary hypertension, which is one of the major complications of SCD, with severe consequences on the right-side heart chambers.^3 , 11 - 17^

Patients with SCD are at increased risk for myocardial ischemia and sudden death, especially with the aging of the affected population.^6 , 11 , 18^ Chest pain is usually attributed to vascular occlusive crisis, and the diagnosis of myocardial infarction is frequently missed, occasionally made only upon autopsy.^18^ Therefore, ischemic heart disease may be present in a significant number of patients with SCD.

Exercise testing has been used widely to detect myocardial ischemia in patients with chest pain syndromes or potential symptom equivalents.^19^ However, the metabolic changes induced by exercise may stimulate erythrocyte sickling and promote vascular occlusions.^20 , 21^ This fact raised a dilemma of either recommended exercise for these patients or deprives them from beneficial effects of physical activity. Although previous studies showed a normal exercise tolerance in SCD patients,^22 , 23^ they had several limitations, including a small number of patients and the use of a six-minute walk test to assess functional capacity. Therefore, there is a lack of evidence to indicate exercise programs for SCD patients. Furthermore, it is unclear whether exercise-induced parameters obtained from symptom-limited exercise tests are associated with adverse outcomes in the SCD setting.

Therefore, in this study, we sought to 1) verify the exercise tolerance in patients with SCD; 2) determine the factors associated with the duration of exercise testing; 3) examine the impact of exercise-induced cardiovascular response on clinical outcome; and 4) assess the feasibility and safety of exercise testing in the population with SCD.

## Methods

### Study population

This was a single center study in which patients with SCD, confirmed by hemoglobin electrophoresis, were prospectively enrolled. Patients who were unable to perform exercise testing due to orthopedic or other organic problems associated with SCD (pain episodes, severe venous insufficiency, cardiovascular or respiratory decompensation) were excluded.

B-type natriuretic peptide (BNP) levels were measured using standard radioimmunoassay in all patients immediately before exercise testing. The research protocol was approved by the Ethics Committee of the Federal University of Minas Gerais and written informed consent was obtained from all patients.

### Exercise testing protocol

Symptom-limited exercise was performed on a treadmill (Centurium 200, Micromed, Brazil), using a modified Bruce protocol, which, in the initial stages, presents smaller increments in the effort load , allowing for better adaptation and tolerance to exercise. This protocol is derived from the standard Bruce protocol and presents 3-minute stages, which are different only in the first stage, which presents normal initial velocity of the first stage original protocol, changing only in the slope (first 3 minutes, without inclination). The second stage is similar to the first stage of Bruce, and, after this it follows the usual protocol. Thus, the relation between workload and O_2_consumption is around 0.5 MET / minute until the third, and thereafter ± 1.2 MET / minute.^19^

A 13-lead ECG was continuously monitored and recorded in each minute, and cuff blood pressure was recorded manually at rest, during the last 30 seconds of each stage and during the 6-min recovery period. After achieving maximal workload, all patients spent 1 minute in a cool-down period at a speed of 2.4 km per hour and a grade of 2.5 percent. After 1 min, all of the patients completed the recovery phase in the supine position.

The test reached the maximal level, with patients remaining on the treadmill until they reached the subjective parameters (dyspnea, fatigue, chest pain or lower limbs, inability to follow the treadmill) of exercise intolerance or usual contraindications for its continuation (such as sustained arrhythmias). The peak VO_2_and METs were estimated at the exercise peak. Presence of ST-T changes, heart rate and blood pressure responses, and arrhythmias were evaluated. Abnormal exercise blood pressure response was defined as either no elevation or increase in systolic blood pressure at peak of exercise < 20 mmHg or a drop in exercise systolic blood pressure below the resting value.^24^ ST-segment changes were considered indicative of ischemia when there was a horizontal or down-sloping ST-segment depression ≥ 1 mm at 60–80 ms after the J point.^19^

Oximetry was performed at rest and during the exercise test using two oximeters: OHMEDA 3800, GE and HELLCOR OXIMAX N-600X, one on each index finger. All exams were performed and analyzed by an experienced cardiologist.

### Echocardiographic evaluation

Echocardiographic assessment was performed according to recommendations of the American Society of Echocardiography,^25^ using a commercially available echocardiograph (GE Vivid Q, Horten, Norway). LV ejection fraction was calculated according to the modified Simpson’s rule, and LV mass was calculated using Devereux’s formula.^26^ Diastolic function was assessed by pulsed-wave Doppler examination of mitral inflow, and by tissue Doppler imaging.^27^ Early diastolic velocity (e’) at the medial and lateral border of the mitral annulus were obtained, and the ratio between peak mitral E and e’ (E/e’) was calculated. Right ventricular function was assessed using peak systolic velocity at the tricuspid annulus by means of tissue Doppler imaging,^28^ tricuspid annular motion, and fractional area change, which was calculated as (RV end-diastolic area – RV end-systolic area)/RV end-diastolic area x 100. Maximal tricuspid regurgitation (TR) velocity was obtained at the 4-chamber or parasternal views. All measurements were performed by a single investigator, blinded to clinical data, and were averaged from 3 beats.

### Endpoint definitions

The primary endpoint was exercise duration, and the secondary endpoint was combined into the following events: (1) death related to SCD, (2) all-cause mortality, (3) three or more acute painful episodes that require hospitalization, (4) acute chest syndrome characterized by a newly pulmonary infiltrate detected by chest radiography associated with chest pain, fever, tachypnea, wheezing, cough, and hypoxemia, and (5) hospitalization for another SCD-related complication, especially a life-threatening infection.

The date of enrollment in the study was defined as the date on which exercise testing was performed. The inclusion period was from August 2015 to September 2016, and the follow-up ended on November 2017. Follow-up data were obtained during clinical follow-up appointment or telephone interviews.

### Statistical analysis

The study was designed to achieve 90% power to detect a 15% prevalence of ECG abnormalities suggested of myocardial ischemia in the overall population with SCD. We assumed that at least 10 patients will have ischemic ST-T abnormalities, returning an estimated sample size of 93.

Categorical data were presented as numbers and percentages, continuous data were expressed as mean ± standard deviation (SD) or median and interquartile range, depending on the pattern of distribution of each variable. Shapiro-Wilk test was performed to evaluate the distribution of the continuous variables.

To determine the factors associated with exercise testing duration, linear regression models with univariate and multivariate analyses were performed. Assumptions for linear regression analysis were verified with no significant violations observed.

Cox regression analysis was performed to determine the characteristics that were independently associated with composite endpoint. Clinical, laboratory, echocardiographic, and exercise testing variables that were clinically relevant or significantly associated with events in the univariate analysis were included in the multivariate logistic regression model. The variables that entered into the final model were age, gender, laboratory (reticulocytes and hemoglobin concentrations), echocardiographic (TR maximal velocity, E/e’ ratio, and LV indexed mass), and exercise testing (abnormal pressure response and presence of ischemia) parameters. A p-value<0.05 was considered to be statistically significant.

Statistical analysis was performed using SPSS, version 22.0 (SPSS Inc., Chicago, Illinois).

## Results

### Clinical characteristics of the study population

A total of 120 outpatients were included, but 7 were unable to perform the exam in an exercise testing room, leaving 113 patients who completed the study protocol. Of these, 71 were carriers of hemoglobin (Hb) SS, 40 HbSC, and 2 with sickle cell-beta zero thalassemia (Hb S-β^0^-thal). The mean age of the patients was 36.2 ± 12.4 years (range, 18-65 years), and 62 patients were women (52%). The majority of the patients are asymptomatic, in NYHA functional class (FC) I (77%), whereas 24 (20%) were in class II and 4 (3%) in class III. The clinical characteristics of the study population are summarized in Table 1 . Sixteen patients (13%) had hypertension, and 43 patients (36%) had renal dysfunction. Hospitalization in the past year occurred in 25 patients (21%), 2 or more times in 11 patients (9%).

**Table 1:** – Baseline Characteristics of the Study Population

Variables*	Value
Body surface area (m^2^)	1.7 ± 0.2
Heart rate (bpm)	75.8 ± 13.6
Systolic/diastolic blood pressures (mmHg)	117.4 ± 14.6/73.2 ± 4.3
Hemoglobin (g/dl)	9.9 ± 2.2
Reticulocytes (% of erythrocytes)	5.6 [3.6/8.7]
Leukocyte count (x10^3^/l)	8.6 ± 3.0
Lactate dehydrogenase (U/l)	575 [413/833]
Aspartate aminotransferase (U/l)	23 [16/32]
Ferritin (ng/ml)	181 [75/388]
Total bilirubin (mg/dl)	1.7 [1.1/3.0]
Creatinine (mg/dl)	0.7 [0.6/0.8]
B-type natriuretic peptide (BNP, pg/ml)	27 [11/62]
**Echocardiographic measurements**	
Left ventricular end-diastolic diameter (mm)	51 [48/56]
Left ventricular end-systolic diameter (mm)	33[30/37]
Left ventricular ejection fraction (%)	63 [59/65]
Indexed LV mass (g/m^2^)	103.2 [85/130]
Peak early diastolic transmitral flow velocity (E,cm/s)	93.1 ± 22.2
Peak late transmitral flow velocity (A, cm/s)	55.9 ± 16.9
Deceleration time (ms)	206.0 ± 41.6
E/A ratio	1.8 ± 0.7
E/e’ ratio †	6.8 ± 2.2
Indexed left atrial volume (mL/m^2^)	42.1 ± 14.8
Right ventricular fractional area changing (%)	44.2 ± 5.8
Right ventricular peak systolic velocity (cm/s)	14.5 ± 3.0
Tricuspid annular motion (mm)	25.9 ± 4.3
Right ventricular myocardial performance index	0.12 ± 0.07
Tricuspid regurgitation maximal velocity‡ (m/s)	2.2 ± 0.3
Right atrial area (cm^2^)	16.2 ± 3.4

*Values are expressed as the mean value ± SD, or median [interquartile range]. e’: early diastolic mitral annular velocity at septal and lateral mitral annulus, E/A: ratio of early to late transmitral flow velocity. † E/e’: ratio of the early diastolic transmitral flow velocity to early diastolic mitral annular velocity (average at septal and lateral mitral annulus). ‡Peak systolic velocity at the tricuspid annulus by tissue Doppler imaging.

Stroke was previously diagnosed in 16 patients (13%), who were under hypertransfusion and were without significant motor sequelae. The most frequently used medications were folic acid (93%), hydroxyurea (62%), and angiotensin-converting enzyme inhibitors or angiotensin receptors blockers (23%). Seven patients (6%) were taking furosemide. All patients who were clinically stable presented mild anemia with hemoglobin levels of 9.9 ± 2.2 g/dl ( Table 1 ). B-type natriuretic peptide concentrations were within the normal range.

The echocardiographic measurements are demonstrated in Table 1 . The majority of the patients had normal ventricular dimensions with preserved systolic function. Left atrial volume was increased, whereas other parameters to assess diastolic function were normal, especially tissue Doppler-derived E/e’ ratio, which was within the normal range. Similarly, right ventricular dimensions and tricuspid regurgitation maximal velocity jet were also within the normal range. Only 2 patients presented a tricuspid regurgitation jet velocity ≥3 m/s.

### Exercise testing

Ischemic ST abnormalities compatible with criteria for ischemia during the effort were detected in 19 patients (17%). Exercise testing characteristics are presented in Table 2 .

**Table 2:** – Patient characteristics during exercise testing

Variables*	Value
Oximetry (%)	95 [92/96]
Peak HR (beats/min)	158.4 ± 21.0
Peak HR (% predicted)	86.9 ± 10.0
Peak VO_2_(ml.Kg^-1^.min^-1^)	31.0 ± 9.7
MET	8.9 ± 2.8
Presence of ischemia	20 (17)
Supraventricular premature beats	40 (33)
Ventricular premature beats	17 (14)
Abnormal blood pressure response	11 (9)
Changes in systolic blood pressure (mmHg) †	27.5 ± 14.9
Delta of systolic pressure/exercise duration (mmHg/min) ‡	3.1 ± 1.5

*Values are expressed as the mean value ± SD, number (percentage) of patients, or median [interquartile range]. † Systolic blood pressure at peak - at rest. ‡ Systolic blood pressure at peak - at rest / exercise time; HR: heart rate; MET: metabolic equivalent for task.

In the overall population, subjective assessment of functional capacity during anamnesis by NYHA functional class (FC) was associated with that measured by exercise testing. Functional capacity was measured in METs, with the mean value of 8.9 ± 2.8, range from 1.5 to 17.3. The patients in class I achieved 9.4 METs whereas those in class III achieved less than 4 METs. The relationship between functional class as assessed by anamnesis and ergometry is shown in Figure 1 .

**Figure 1:** – Association between functional capacity by NYHA functional class assessed by anamnesis and exercise capacity measured by ergometry.

Supraventricular premature contractions were frequent during exercise, isolated in 16% of the cases, and complexes with some episodes of paroxysmal supraventricular tachycardia in 17% of the patients. Isolated ventricular premature contractions occurred in 14 patients (12%). Abnormal blood pressure response was found in 10 patients (9%), with a mean increase of systolic blood pressure of 14 mmHg, when compared to those with anormal response, in whom the mean increase of blood pressure was 29 mmHg (p=0.002). Following the exercise testing, within 48 hours, two patients (1.8%) experienced pain crises that required hospitalization for treatment.

### Factors associated with exercise duration

In the overall population, the exercise duration was 9.2 minutes, ranging from 1.1 to 15.5 minutes. Several clinical, laboratory, and echocardiographic variables were tested for a possible association with exercise tolerance ( Table 3 ). The potential predictors that were selected for the multivariate model were age, gender, oximetry at rest, hemoglobin concentration, and echocardiographic parameters of LV diastolic function, RV function, and pulmonary pressure assessed by TR maximal velocity. TR maximal velocity and E/e’ ratio were the main factors associated with exercise time in the univariate analysis. In the multivariate linear regression analysis, including the laboratory markers of disease severity, TR maximal velocity and E/e’ ratio emerged as important factors associated with exercise duration, after adjustment for age and gender ( Table 4 ).

**Table 3:** – Factors associated with exercise time

Variables	Univariate		Multivariate	

	Beta	p-value	Beta	p-value
Age (years)	-0.067	0.001	-0.038	0.045
Male gender	1.386	0.003	1.195	0.006
Beta-blockers	-2.158	0.014	…	…
Leg ulcers	-1.242	0.034	…	…
Previous stroke	-1.475	0.042	…	…
Indexed LA volume (mL/m^2^)	-0.049	0.002	…	…
Peak A velocity (cm/s)	-0.032	0.025	…	…
Deceleration time (ms)	-0.017	0.004	…	…
E/e’ ratio	-0.358	<0.001	-0.224	0.018
TR maximal velocity (m/s)	-2.675	<0.001	-1.810	0.015
Indexed LV mass (g/m^2^)	-0.015	0.014	…	…
Systolic blood pressure (mmHg)	-0.045	0.005	…	…
Oximetry (%) at rest	0.240	0.022	…	…
B-type natriuretic peptide (pg/ml)	-0.006	0.001	…	…
Hemoglobin (g/dl)	0.387	<0.001	…	…
Ferritin (ng/ml)	-0.001	0.001	…	…
Lactate dehydrogenase (IU/l)	-0.002	0.004	…	…
Proteinuria	1.436	0.005	…	…

*LA: left atrial; LV: left ventricular; TR: tricuspid regurgitation.*

**Table 4:** – Cox proportional-hazards analysis for predicting adverse outcomes in patients with sickle cell disease

Variables	Univariate		Multivariate	

	HR (95% CI)	p-value	HR (95% CI)	p-value
Genotype Hb SS	2.546 (1.020-6.351)	0.045	…	…
Hemoglobin (g/dl)	0.803 (0.664-0.970)	0.023	0.688 (0.552-0.858)	0.001
LV mass (g/m^2^)	1.007 (1.000-1.015)	0.055	…	…
LAV (mL/m^2^)	1.022 (0.999-1.046)	0.060	…	…
Right atrial area (cm^2^)	1.143 (1.042-1.255)	0.005	…	…
TAM (mm)	1.098 (1.008-1.197)	0.033	…	…
TR velocity (m/s)	3.729 (1.474-9.433)	0.005	…	…
Peak A velocity (cm/s)	0.976 (0.955-0.998)	0.031	0.964 (0.933-0.997)	0.034
Abnormal SBP response	4.110 (1.346-12.550)	0.013	4.990 (1.316-18.921)	0.018
BNP (pg/ml)	1.001 (1.000-1.003)	0.052	…	…

*CI: confidence interval; HR: hazard ratio; LAV: left atrial volume; LV: left ventricular; SBP: systolic blood pressure; TAM: tricuspid annular motion; TR: tricuspid regurgitation; BNP: B-type natriuretic peptide.*

### Predictor of adverse events

During a mean follow-up of 10.1 months (range, 1.2 to 26), the endpoint was reached in 27 patients (23%): 4 patients died (one death was unrelated to SCD), 8 were hospitalized due to ≥ 3 acute painful episodes, 11 had acute chest syndrome, and 4 were hospitalized with other SCD-related complications.

Several variables were tested for a possible association with an adverse outcome ( Table 4 ). The potential predictors that were selected for the multivariate model were genotype Hb SS, Hemoglobin levels, left ventricular mass, left atrial volume, right atrial area, tricuspid regurgitation peak velocity, peak transmitral A velocity, BNP levels, and abnormal blood pressure response to exercise. In the multivariate analysis, the independent predictors of adverse events were hemoglobin concentration, peak transmitral A velocity, and abnormal blood pressure response to exercise. The cumulative incidence of adverse events by systolic blood pressure response is shown in Figure 2 .

**Figure 2:** – Cumulative incidence of adverse events in patients with SCD who presented abnormal blood pressure response to exercise as compared to those with a physiological response (p-value of 0.027).

## Discussion

This study sought to provide some information on exercise tolerance in SCD patients. As there is a lack of evidence in the literature about exercise testing in SCD, our results show that exercise testing in chronic compensated patients with SCD is feasible, relatively safe, and can be performed in a hospital environment with an experienced team. Moreover, exercise testing provides useful information for the management of patients with SCD.

There is a lack of evidence to indicate an exercise program for patients with SCD. The major question faced by healthcare professionals involved in SCA management is the safe level of physical exercise they should recommend for their patients.^21^

As physical activity is known to induce metabolic changes that can potentially precipitate a vaso-occlusive crisis, patients are usually encouraged to exercise on a symptom-limited basis. The presence of anemia induces a faster transition from aerobic to anaerobic metabolism during exercise, which may stimulate the polymerization of hemoglobin S and promote microvascular occlusions.^29 , 30^ Additionally, the dehydration that occurs during exercise, associated with the acute episodes of tissue hypoxia, may also contribute to the sickling of the red blood cells. Therefore, although our study and others have demonstrated relative safety of physical activity in SCD,^21^ it is not risk-free. We observed two complications after the test, reinforcing the need for medical care, including hydration, to perform exercise testing in this vulnerable population.

However, recent evidence suggests that SCD patients may practice physical activities even if specific recommendations about exercise duration and intensity are needed.^21 , 30^

The presence of arrhythmias during exercise varies greatly in the literature. In our study, 16% of the patients presented supraventricular arrhythmias, which is higher than expected for this group of patients.^19 , 31^ This is probably due to left atrial enlargement and diastolic dysfunction often seen in SCD, which are the main factors associated with these arrhythmias,^32^ adjusted by age. The prevalence of ventricular arrhythmias was similar to data from the literature.^19^ The presence of ischemic changes of the ST-segment, suggesting that myocardial ischemia is considered frequent in SCD, ranging from 10-50%.^6 , 11 , 33^ We found a prevalence of 17%, with no other findings indicating obstructive coronary disease.

### Determinants of exercise tolerance in patients with SCD

Accentuated impairment in exercise capacity has consistently been found in SCD patients. Several factors contribute to exercise intolerance, including possible cardiac filling abnormalities, chronic anemia, pulmonary vascular disease, peripheral vascular disease related to microvascular occlusion.^11 , 21 , 34 , 35^ Three main mechanisms for exercise limitation in SCA were proposed: anemia, pulmonary vascular disease, and peripheral vascular disease and/or myopathy.^21^ Indeed, in our study, the tricuspid regurgitation velocity that estimates pulmonary artery systolic pressure remained as an important determinant of exercise duration after adjustment for age and gender. Similarly, a tissue Doppler-derived E/e’ ratio, which is a marker of high LV filling pressure was an independent factor associated with exercise duration.

In agreement with our findings, a previous study showed that a reduction in the 6-min walk distance was independently associated with echocardiographic measures of pulmonary hypertension, expressed by tricuspid regurgitation velocity, and with measures of diastolic dysfunction, suggesting two major independent determinants of exercise intolerance.^36^

In the general population, abnormalities of left ventricular diastolic function, measured by E/e’ ratio, are independently associated with exercise capacity.^37^ Although males had a greater exercise capacity than females, the magnitude of this difference decreased with age. Compared to those with normal diastolic function, patients with mild diastolic dysfunction (impaired relaxation) had a progressive increase in the magnitude of reduction in exercise capacity with advancing age.^37^ In the present study with asymptomatic patients with mild diastolic dysfunction, age was inversely correlated with exercise capacity.

### Abnormal blood pressure response and adverse outcomes in SCD

The mean arterial pressure should normally increase by near 40% during incremental exercise as a result of the increase in cardiac output, with a progressive increase in systolic blood pressure.^24^ Abnormal blood pressure responses are relatively common, and their potential clinical value has increasingly drawn attention.^38^ Although difficult to determine on the basis of varying definitions, the prevalence of exercise hypotension has been reported in up to 6%.^39^

Exercise-induced hypotension has long been considered a poor prognostic sign in those with established cardiovascular disease.^40 - 42^ A systematic review and meta-analysis showed that a hypotensive response predicts longer-term fatal and non-fatal cardiovascular events and all-cause mortality.^43^ This was observed irrespective of disease presentation, mode of exercise undertaken, intensity of exercise, or how exercise hypotension was defined. In agreement, we found that abnormal blood response was an independent predictor of adverse events, after adjustment for well-known prognostic factors.

Several mechanisms have been proposed to explain the association between the increased risk of adverse cardiovascular outcomes and an insufficient rise, or drop, in blood pressure during incremental exercise testing.^38 , 42^ During exercise, decreased systolic blood pressure below resting values has been linked to underlying cardiovascular disease, including left ventricular dysfunction, coronary artery disease, and aortic outflow obstructions.^42 , 43^ Abnormalities in the autonomic nervous system during exercise testing are likely observed in patients who appear with decreased systolic blood pressure responses. Autonomic imbalance has been related to the development of heart failure, and similar disturbances possibly occur in those with decreased exercise systolic blood pressure response.^44^ A previous study showed that even modest elevations in systolic blood pressure during exercise stress testing are associated with a decreased risk of all-cause death and myocardial infarction.^42^ However, the etiology of exercise-induced hypotension is multifactorial and complex.

In the setting of SCD, systemic blood pressure is reported to be lower in SCD patients without comorbidities, when compared to the general population.^45^ SCD patients with blood pressure values above the expected range for this population – “relative systemic hypertension” – had increased risk of stroke and death.^46^ . The exact mechanism by which exercise induced abnormal blood pressure response in SCD patients is related to adverse outcomes needs to be defined. Myocardial ischemia induced by exercise may cause left ventricular dysfunction. Indeed, a previous study reported that left ventricular end-diastolic volume decreased most markedly with exercise in patients exhibiting ischemic ECG.^47^ On the other hand, another investigation found that the patients who had ischemic responses when exercising also showed an elevated double product (systolic blood pressure x heart rate) with an excessive elevation in blood pressure, suggesting increased myocardial oxygen demand during exercise in this population.^48^

Pulmonary hypertension is also associated with exercise limitation and poor prognosis in SCD patients.^21^ Although in our study the pulmonary pressure response to exercise was not assessed, its excessive elevation during exercise may contribute to right ventricular dysfunction, reduction in cardiac output, with consequent hypotensive response to exercise. Indeed, the relationship between adverse outcome and abnormal blood pressure response to exercise in SCD patients is complex, likely mediated by chronic complications, including anemia, pulmonary vascular disease, and left ventricular diastolic dysfunction.

### Study limitations

The study has some limitations. The sample size was estimated to detect ECG abnormalities related to myocardial ischemia in SCD, which limits the analysis regarding the predictors of adverse events. The patients enrolled in this study are referred from an outpatient clinic, including a wide spectrum of the SCD, but with a small number of more severe disease subgroups, particularly with pulmonary hypertension, which limits its external validity.

A total of 34% of the patients had an SC subtype, which limited our conclusions for the entire population of SCD. In addition, the use of the cardiopulmonary exercise test would be the ideal tool to study the determinants of functional capacity in these patients. Another limiting factor is related to the measurement of blood pressure during exercise. It is well described the difficulty of this measurement during physical activity, which may compromise the reproducibility of our finding.

## Conclusions

Exercise testing in SCD patients who were clinically stable is relatively safe and feasible, providing valuable clinical information, and may be helpful in aerobic conditioning. Exercise-induced ischemic electrocardiographic changes were frequent, whereas pain crises after exercise were uncommon. The main determinants of exercise duration were left ventricular diastolic function and pulmonary artery pressure estimated by tricuspid regurgitation velocity. Abnormal blood response was an independent predictor of adverse events. Further studies are needed to determine the safety of the exam in larger samples, together with the underlying mechanisms associated with the increased risk of adverse events in patients with SCD with decreased systolic blood pressure response during exercise stress testing.
